# A Rare, Late Complication after Automated Implantable Cardioverter-Defibrillator Placement

**Published:** 2004-10-01

**Authors:** Michael Shapiro, Sam Hanon, Paul Schweitzer

**Affiliations:** Department of Cardiology, Beth Israel Medical Center, University Hospital and Manhattan Campus for the Albert Einstein College of Medicine, New York, USA

**Keywords:** pacemaker complication, skin erosion, extrusion

## Abstract

This article describes an interesting case of automated implantable cardioverter defibrillator (AICD) extrusion fifteen months after implantation.  The case report is followed by a discussion of the causes and treatment of skin erosion following pacemaker/AICD insertion.

## Case report

 The patient is a 65 year old man with a history of chronic renal insufficiency, dyslipidemia, hypertension, peripheral arterial disease, and coronary artery disease status post myocardial infarction in 1994.  He was revascularized with 3-vessel coronary artery bypass grafting on 3/12/02 after he developed unstable angina.  His post-operative course was complicated by a deep venous thrombosis. Treatment was initiated with warfarin and the patient was subsequently discharged from the hospital.  He was readmitted three weeks later with mild shortness of breath.  A ventilation/perfusion scan was negative for pulmonary embolism.  However, while on telemetry, the patient had an episode of non-sustained ventricular tachycardia.  He underwent diagnostic electrophysiologic testing at which time he was inducible for ventricular tachycardia.  On 4/2/02, an AICD was implanted.  He was discharged without incident and did well for over a year without any arrhythmic event requiring ICD shock. One week prior to the current admission the patient noted a skin tear over the AICD with protrusion of the device to the anterior chest wall.  He visited his cardiologist to evaluate the extrusion of the device at which time he was immediately referred for hospital admission.

The patient admitted to minimal skin tenderness over the site but denied bloody or purulent drainage, fevers, chills, or malaise.  His medications included  metoprolol XL 50 mg daily, aspirin 81 mg daily, clopidogrel 75 mg daily, atorvastatin 10 mg daily, isosorbide mononitrate 20 mg daily, and gabapentin 300 mg daily.  Social history, family history, and review of systems were non-contributory.

**Figure 1:**
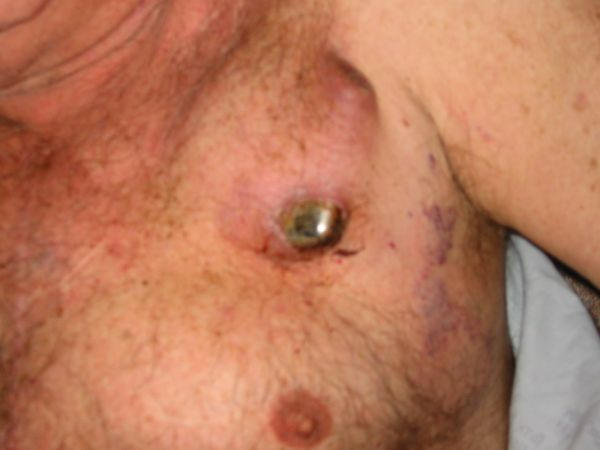
Erosion of the AICD through the skin. Note upper right corner of subcutaneous pocket shows severe erythema with signs of pre-erosion.

**Figure 2:**
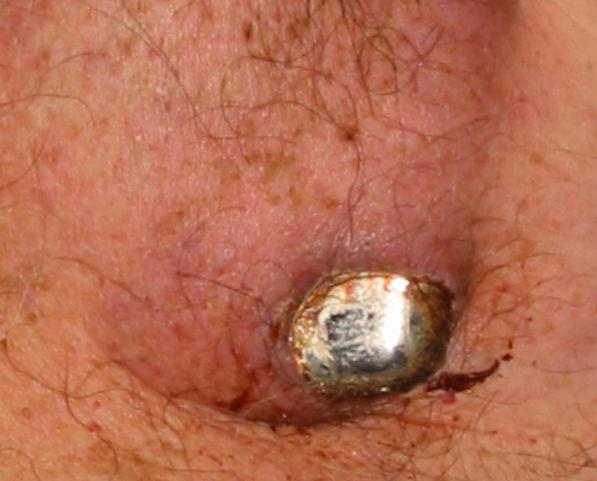
Close up examination of AICD eroding through the skin.

On physical examination, the patient had a temperature of 97.8° F, blood pressure 122/78 mm Hg, pulse 86 beats per minute and regular, and respiratory rate of 20 breaths per minute.  He was a well developed, well nourished male sitting comfortably in no distress.  Examination of his neck revealed no JVD at 30°, normal carotid upstrokes without bruits, and no thyromegaly.  His chest was clear and heart examination revealed a non-displaced point of maximal impulse with a normal apical impulse, normal S1 and S2 without an S3 or S4.  His pulse was regular with an apical 2/6 holosystolic murmur with radiation to the axilla.  His anterior chest wall revealed a well healed sternotomy scar with erythema in the left supraclavicular area at the point where his AICD was extruding from his skin without drainage (see Figure 1 and Figure 2).  His abdomen was obese but soft without hepatomegaly.  Neurologically, he was grossly intact.  His lower extremities revealed no edema and distal pulses were intact.  His initial laboratory results were normal including metabolic profile, complete blood count with differential, and coagulation profile.  His chest radiograph revealed clear lungs, normal heart size, and a dual chamber pacemaker/defibrillator with leads in proper position.  His electrocardiogram (ECG) revealed a normal sinus rhythm with right bundle branch block, left posterior fascicular block, and an old inferior infarct.  This ECG was unchanged from prior tracings.  The patient was admitted to the hospital and three sets of blood cultures were obtained. He was subsequently started on vancomycin 1 g intravenously q 12h. The following day his device was explanted and the pocket debrided.  Blood cultures as well as cultures from the device and surrounding skin and subcutaneous tissue revealed no growth.  He was continued on antibiotics and discharged from the hospital without complications.  The discharge plan was to replace the AICD at a future date, upon healing of the operative site.

## Discussion

This case illustrates an unusually severe form of pacemaker/AICD skin erosion.  This rare complication has been previously described in the literature.  However, prior to the study performed by Kiviniemi et al [1], most of the existing data was based on information from the 1970s [2]. This more recent investigation retrospectively analyzed four hundred forty-six patients who received permanent pacemakers and reported the complications noted during implantation or follow-up.  Pacemaker erosion was detected in 0.9% of the patients.

Two main culprits have been implicated in the pathogenesis of pacemaker extrusion. First, infection of the site can lead to skin erosion [3]. Obviously, prevention of and monitoring for infection is paramount to averting this process. DaCosta et al. performed a meta-analysis of antibiotic prophylaxis for pacemaker implantation [4].  They concluded that antibiotics administered during the peri-implant period reduced the incidence of infective complications following pacemaker implantation, including short-term pocket infection, skin erosion, or septicemia.

The other cause of skin erosion is pressure necrosis of the overlying tissue and skin [3].  Gross pacemaker extrusion is usually signaled by a preceding period of “ pre-erosion,” during which there is discomfort and discoloration of thinning tissue tensely stretched over a protrusion of the pacing apparatus ( Figure 2).  Griffith et al. concluded that if pacemaker erosion is not caused by infection it can be successfully managed by ipsilateral re-implantation, a financially advantageous solution [5]. Identification of pre-erosion allows salvage of the pacing system, as the hardware can be repositioned under the pectoralis muscle or in an abdominal location.

Risk factors for skin erosion include factors related to the device itself as well as to the implantation site. The mass and configuration of the pacemaker as well as the need for extra hardware (e.g., lead adaptor) in the pocket may lead to local tension and pressure necrosis. Additionally, precise surgical construction of the pacemaker pocket is vital. A pocket of inadequate size or a paucity of subcutaneous tissue may contribute to local complications. The pocket plane should be created on the surface of the muscle since superficial pockets lend themselves to erosion [6].

If true erosion occurs, the system is considered contaminated and current opinion favors removal of the generator and leads.  Extensive debridement of the pocket and prolonged irrigation and antibiotic therapy may provide an alternate option to removal in cases of both erosion and frank infection [7], but this approach is not generally preferred.  Attempts have been made to preserve the implanted leads by debriding locally and severing the pacemaker leads while leaving them in situ. However, given the relative safety and efficacy of percutaneous lead removal, complete removal of the leads is the ideal approach if sterility is questioned.
